# Serum 25-Hydroxyvitamin D Concentration as a Biomarker and Immunomodulator in Patients with Acute Ischemic Stroke: A Retrospective Single-Center Study

**DOI:** 10.3390/nu18132179

**Published:** 2026-07-04

**Authors:** Milena Świtońska, Agnieszka Rogalska, Alicja Szulc, Oliwia Jarosz, Magdalena Konieczna-Brazis, Łukasz Wołowiec, Piotr Płeszka, Krzysztof Tojek, Jacek Budzyński

**Affiliations:** 1 Department of Neurology and Clinical Neurophysiology, Ludwik Rydygier Collegium Medicum in Bydgoszcz, Nicolaus Copernicus University in Toruń, 85-168 Bydgoszcz, Poland; m.switonska@cm.umk.pl (M.Ś.); alicja.szulc@cm.umk.pl (A.S.); magda.brazis@cm.umk.pl (M.K.-B.); 2 Jan Biziel University Hospital No. 2 in Bydgoszcz, Ludwik Rydygier Collegium Medicum in Bydgoszcz, Nicolaus Copernicus University in Toruń, 75 Ujejskiego Street, 85-168 Bydgoszcz, Poland; agnieszka.rogalska@biziel.pl (A.R.); lukasz.wolowiec@cm.umk.pl (Ł.W.); piotr.pleszka@biziel.pl (P.P.);; 3 Doctoral School of Medical and Health Sciences, Ludwik Rydygier Collegium Medicum in Bydgoszcz, Nicolaus Copernicus University in Toruń, 85-168 Bydgoszcz, Poland; 503671@doktorant.umk.pl; 4 Department of Cardiology and Clinical Pharmacology, Faculty of Health Sciences, Ludwik Rydygier Collegium Medicum in Bydgoszcz, Nicolaus Copernicus University in Toruń, 85-168 Bydgoszcz, Poland; 5 Department of General and Minimally Invasive Surgery, Faculty of Medicine, Ludwik Rydygier Collegium Medicum in Bydgoszcz, Nicolaus Copernicus University in Toruń, 85-168 Bydgoszcz, Poland; 6 Department of Vascular and Internal Medicine, Ludwik Rydygier Collegium Medicum in Bydgoszcz, Nicolaus Copernicus University in Toruń, 85-168 Bydgoszcz, Poland

**Keywords:** acute ischemic stroke, vitamin D, neuroinflammation, prognosis

## Abstract

***Background***: Several studies reveal an inverse relation between serum 25-hydroxyvitamin D [25(OH)D] concentration and the risk of acute ischemic stroke (AIS). The aim of this study was to determine relationships between 25(OH)D concentration and the course and outcomes of AIS treatment and the level of indices of inflammatory response to brain injury. ***Patients and Methods:*** Retrospective analysis of medical documentation of 1381 real-world AIS patients hospitalized in a single center between 1 January 2020 and 31 May 2025. Serum 25(OH)D level, several inflammatory indices, and clinical data were assessed. ***Results***: Compared to patients in the lowest quartile of 25(OH)D concentration, those in the highest quartile had a shorter length of in-hospital stay, a lower risk of all-cause death, and a lower score for disability on a modified Rankin scale (mRS). Along with an increase in 25(OH)D quartiles, we found: a decrease in neutrophil count; a decrease in glucose, HbA1c, albumin, C-reactive protein (CRP), and CRP-to-albumin, -lymphocyte, -neutrophil, and -platelet ratios; lower neutrophil-to-lymphocyte and -albumin ratios, and lower systemic immune inflammation, and systemic inflammation response indices. In multifactorial logistic regression, the quartile of 25(OH)D (OR, 95% CI: 1.52, 1.09–2.12; *p* = 0.012) was the only variable to have a positive association with a mRS score ≤ 2 at discharge from hospital, and neutrophil-to-lymphocyte ratio, age, diabetes, and treatment with endovascular mechanical thrombectomy were biomarkers of poor functional status at discharge. ***Conclusions:*** Higher 25(OH)D concentration in AIS patients is related to better survival and a lower level of inflammatory response indices and disability at discharge.

## 1. Introduction

Acute ischemic stroke (AIS) is one of the most prevalent causes of cardiovascular mortality and functional impairment [1]. For the last ten years and more, the effectiveness of AIS treatment has improved by virtue of the introduction of reperfusion therapy, which consists of intravenous thrombolysis (IVT), endovascular mechanical thrombectomy (EMT), or both. However, in 30–50% of patients who undergo brain reperfusion therapy, in-hospital mortality or short- and long-term functional status does not improve. This phenomenon is known as “futile recanalization” [2]. Among the potential causes of futile recanalization and overall poor prognosis in AIS patients are: hemorrhagic transformation, hospital-acquired infection (HAI), and in most cases a sub-form of pneumonia or urinary tract infection, as well as the individually varying severity of systemic and local inflammatory responses to brain injury, and comorbidities [2,3,4]. In recent years, several observational studies have been published on the role of low serum 25-hydroxyvitamin D [25(OH)D] concentration (<20–50 ng/mL) as a biomarker of cerebrovascular disease risk (e.g., AIS and Parkinson’s disease), as well as a biomarker of poor short- and long-term prognosis in patients with cerebrovascular disease, including AIS, in regard to in-hospital mortality, stroke severity, and stroke recurrence; functional and dependence status; post-stroke cognitive functions, dementia, and depression; post-stroke acute kidney injury; and musculoskeletal consequences of AIS [5,6,7,8,9,10,11,12,13,14,15,16,17,18,19,20,21,22,23,24,25,26]. Li et al. [20] and Pan et al. [24] describe reverse J-shaped relationships between serum 25(OH)D concentration and AIS occurrence and recurrence. However, not all studies, including genetic analyses, corroborate a causal relationship between serum 25(OH)D concentration and cause-specific mortality outcomes in patients with coronary artery disease and stroke [25,26]. Moreover, the majority of interventional, randomized studies and meta-analyses on the effectiveness of vitamin D supplementation (600–3225 international units [IU]/day) have not found neuroprotective activity from vitamin D [1,5,24,27,28,29,30,31,32,33,34]. In a study by Hesami et al. [34], only intramuscular administration of a single 600,000 IU dose of vitamin D exerted neuroprotective effects in patients with moderate ischemic stroke, measured using a National Institute of Health Stroke Scale (NIHSS) score of 5–15, according to favorable effects on functional clinical outcomes after a three-month follow-up, but without significant influence on serum neuron-specific enolase level.

It is known that vitamin D regulates calcium and phosphate metabolism, immunological resistance, skeletal muscle metabolism and reconstruction, as well as anti-cancer resistance. As vitamin D acts as a provitamin, its clinical effects depend on liver and kidney functions, which enable the double-hydroxylation and activation of vitamin D [5], but its general health state is very important too. Among the potential pathomechanisms of the neuroprotective action of vitamin D and inverse relationships between serum 25(OH)D concentration and the risk and course of AIS are: an influence in brain development and function; immunomodulation and neuroinflammation; stimulation of synaptic and neuroplastic changes; maintenance of blood–brain barrier integrity; reduction in oxidative stress; effect on homocysteine and brain-derived neurotrophic factor levels; and improvement in skeletal muscle mass, strength, and performance (post-stroke sarcopenia risk reduction) [1,5,25,26]. Moreover, vitamin D modulates cardiomyocyte calcium handling, endothelial function, vascular smooth muscle proliferation, vascular remodeling and inflammation (enhancement of anti-inflammatory cytokines), arterial stiffness, blood pressure and renin–angiotensin–aldosterone system activity, as well as regulating platelet aggregation and thrombotic risk through its influence on thrombomodulin and tissue factor expression [31,35,36,37,38,39]. Vitamin D deficiency may also augment the risk of AIS by its association with atrial fibrillation prevalence by 1.8–1.94 times, depending on the severity of the deficiency [37,38]. It is known that atrial fibrillation increases the risk of cardioembolic stroke by 4–5 times, which may, in turn, explain the results of Mendelian randomization analysis by Habibi et al. [39] which revealed that increased 25(OH)D levels may play a protective role against cardioembolic stroke.

Taking into account the potential neuroprotective activity of vitamin D and its prognostic importance presented above, we performed a retrospective analysis of the single-center medical documentation of AIS patients treated in a university hospital, in order to assess the relationships between serum 25(OH)D concentration and the short-term course and outcomes of AIS treatment. We did so in order to highlight the importance of the real-world assessment of serum 25(OH)D concentration as a biomarker that could be helpful in the stratification of AIS severity and the personalization of patient management. Moreover, in our study, we conducted our analysis to identify relationships between serum 25(OH)D concentration and the level of inflammatory response indices for brain injury [3,4]. To the best of our knowledge, this is the first study to address this gap in knowledge, revealing a potential pathomechanism of neuroprotective vitamin D activity through the regulation of neuroinflammation.

## 2. Patients and Methods

### 2.1. Patients

We retrospectively analyzed the medical documentation of 1381 patients admitted to a single-center university hospital due to AIS between 1 January 2020 and 31 May 2025. The inclusion criteria were as follows: diagnosis of AIS (code I63, according to the International Classification of Diseases, 10th Revision [ICD-10]) treated both conservatively and with the intention of achieving brain reperfusion using EMT, preceded or not by IVT, with a recombinant tissue plasminogen activator (rt-PA) (alteplase: 0.9 mg/kg of body weight; maximum 90 mg per one hour); age ≥ 18 years; and determination of serum 25(OH)D concentration on admission. The following exclusion criteria were applied: known supplementation of vitamin D (in medical history obtained and in patient medication list), signs of clinical infection on admission, hemorrhagic stroke and transient ischemic attack as a reason for admission, and history of leukemia or another type of neoplasm. The diagnosis of AIS was made by an experienced neurologist on duty and confirmed using computed tomography angiography. Comorbidities, atherosclerotic cardiovascular disease risk factors (e.g., hypertension, diabetes mellitus, atrial fibrillation, hypertension, chronic coronary syndrome, and chronic cardiac failure), occurrence of the outcomes measured, and HAI were identified on the basis of the data available from the medical documentation. EMT was performed by an experienced, certificated interventional radiologist, and additional AIS patient management, pharmacotherapy, and physiotherapy were applied in accordance with the current Polish recommendations. NIHSS and modified Rankin scale (mRS) scores were assessed within 24 h of admission, and at discharge. The presumed etiological mechanism of AIS was classified according to the Trial of Org 10172 in Acute Stroke Treatment (TOAST) classification, distinguishing the following stroke subtypes: large-artery atherosclerosis (LAA) plaque buildup, cardioembolic stroke (CE), small-vessel occlusion (SVO)/lacunar stroke, and stroke of undetermined etiology (SUE).

### 2.2. Methods

Retrospective analysis of the medical documentation of all consecutive patients hospitalized in the Neurology Department of a university hospital due to AIS was conducted on the basis of the primary diagnosis at discharge (under ICD-10 code I63) between 1 January 2020 and 31 May 2025. We obtained clinical and laboratory data measured using standard methods at a certified central hospital laboratory (the first measurement was taken during hospitalization). The data were as follows: blood smear morphology of white blood cells (leukocytes); and serum vitamin D [as 25(OH)D], C-reactive protein (CRP), albumin, total, high-density lipoprotein (HDL), and low-density lipoprotein (LDL) cholesterol, triglycerides, glucose, hemoglobin A1c (HbA1c), and creatinine concentrations. Serum 25-hydroxyvitamin D [25(OH)D] concentration was measured using electrochemiluminescence immunoassay (Elecsys Vitamin D total III) produced by Roche Diagnostics GmbH, Mannheim, Germany.

The following single parameters were used as biomarkers of inflammatory response to AIS: blood CRP and albumin concentrations; leukocyte and platelet absolute counts; and leukocyte differentials: neutrophils, lymphocytes, eosinophils, and monocytes in absolute counts. We also calculated composite inflammatory response indices: CRP-to-albumin, CRP-to-lymphocyte, CRP-to-neutrophil, CRP-to-monocyte, CRP-to-platelet, and CRP-to-HDL-cholesterol ratios; neutrophil-to-lymphocyte ratio (NLR), neutrophil-to-platelet ratio, and neutrophil(count)-to-albumin ratio; neutrophil percentage-to-albumin ratio (NPAR), lymphocyte-to-monocyte ratio (LMR) and lymphocyte-to-albumin ratio; platelet-to-lymphocyte ratio (PLR), platelet-to-albumin ratio, and platelet-to-hemoglobin ratio (PHR); and monocyte-to-HDL cholesterol ratio [3,4]. Inflammatory Burden Index (IBI) was calculated according to the following formula: IBI = CRP (mg/dL) × NLR; CRP–albumin–lymphocyte (CALLY) index was calculated using the formula: CALLY = [albumin (mg/dL) × lymphocyte count (G/L)]/[CRP (mg/dL) × 10]; lymphocyte–albumin–neutrophil (HLAN) index was calculated according to the formula: HLAN = hemoglobin (g/L) × lymphocytes (G/L) × albumin (g/L)/neutrophils (G/L)/100; hemoglobin–albumin–lymphocyte–platelet (HALP) index was calculated using the following: HALP = hemoglobin (g/L) × albumin (g/L) × lymphocytes (G/L)/platelets (G/L); Systemic Inflammation Response Index (SIRI) was calculated according to the formula: SIRI = neutrophil count × monocyte count/lymphocyte count; Systemic Immune-Inflammation Index (SII) was calculated according to the formula: SII = neutrophil count × PLR; Naples Prognostic Score (NPS) was calculated according to scored cut-offs for albumin, total cholesterol, NLR, and LMR; and The Nutritional Risk Index (NRI) was calculated according to the formula NRI = (1.519 × serum albumin [g/dL]) + (41.7 × actual body weight [kg]/ideal body weight [kg]) [4].

### 2.3. Outcomes Measured

The following outcomes were included in the analysis: in-hospital all-cause mortality; readmission within 14, 30, and 365 days of discharge; length of in-hospital stay (LOS); and scores on neurological patient disability and dependence scales: IHSS and mRS on admission and at discharge, the delta of their scores at discharge and on admission, as well as the percentage of patients with an mRS score ≤ 2 (i.e., 0–2) at discharge.

### 2.4. Bioethics

The anonymized medical documentation of AIS patients who met the inclusion criteria and had none of the exclusion criteria was analyzed retrospectively. This study was not required to be registered as a clinical trial. The investigation was conducted in compliance with the Declaration of Helsinki for medical research. The study protocol was approved by the local, independent ethics committee on human experimentation (Bioethics Committee of Nicolaus Copernicus University in Toruń by the Ludwik Rydygier Collegium Medicum in Bydgoszcz; approval No. KB 376/2025 on 25 June 2025). In accordance with Polish regulations, including the ruling issued by the Bioethics Committee, patients’ informed consent was not required for retrospective analyses of anonymized medical documentation.

### 2.5. Statistics

Statistical analysis was conducted using the licensed version of the statistical software Statistica, version 13.3, developed by Tibco Software, Inc. 2017 (Palo Alto, CA, USA; San Ramon, CA, USA). The normal distribution of the study variables was checked using the Kolmogorov–Smirnov test. Depending on the distribution of the variables, the results were presented as the mean ± standard deviation, n, %, and median, and interquartile range. The statistical significance of differences between groups was verified using unifactorial variance analysis (ANOVA) with the Bonferroni post hoc test and the Chi^2^ test (normally distributed variables) or using the non-parametric Kruskal–Wallis ANOVA test. Degrees of freedom (d.f.) for F or Chi^2^ tests were given in parentheses. Receiver operating characteristic (ROC) analysis was used to determine the predictive value of vitamin D. The statistical significance level was set at a *p*-value of <0.05. Pearson’s linear regression or Spearman’s rank correlation was determined depending on the type and distribution of the variables. Univariate and multivariate logistic regressions were used to determine the adjusted and independent effect of 25(OH)D level on the occurrence of the outcomes measured. To identify confounders, before the multiple regression analysis we compared patients who had achieved a score of 2 on the mRS and those who had not.

## 3. Results

In our retrospective analysis of medical documentation of 1381 AIS patients, we found that those who died during hospitalization and those who were readmitted to the Neurology Department within one year of AIS had significantly lower serum 25(OH)D concentration than those patients who survived hospitalization (17.58 ± 13.27 vs. 21.76 ± 15.61 ng/mL, *p* < 0.01) and who were not readmitted due to stroke recurrence during the 365 days after discharge (21.06 ± 14.28 vs. 22.81 ± 15.28 ng/mL, *p* = 0.010). However, patients in the lowest quartile of serum 25(OH)D concentration were also the oldest and had the highest prevalence of comorbidities (Table 1). We did not find any statistically significant differences in serum 25(OH)D concentration between patients who were readmitted within 14 and 30 days after discharge, and between those who were treated medically and those who were managed with the intention of brain reperfusion (EMT ± IVT). With regard to the TOAST classification of presumed AIS etiology, the study included 432 patients (31.3%) with LAA, 443 (32.0%) with CE, 172 (12.5%) with SVO, and 334 (24.2%) with SUE. Serum 25(OH)D concentration did not differ significantly between the TOAST categories (22.0 ± 15.8 vs. 20.8 ± 15.3 vs. 21.5 ± 14.4 vs. 21.5 ± 16.0 ng/mL, respectively; ANOVA, F(3) = 0.433; *p* = 0.729). Moreover, although the overall chi-square (χ^2^) test showed a statistically significant association between TOAST category and 25(OH)D quartile, only a single statistically significant differences were found across the etiological stroke categories and 25(OH)D quartiles (Table 1).

Next, we analyzed differences between AIS patients who were divided in relation to serum 25(OH)D concentration quartiles: Q1: <10.16; Q2: 10.16–17.77; Q3: 17.78–29.00; and Q4: ≥29.01 ng/mL (Table 1). We found a statistically significant ANOVA F test for differences in 19 of 29 clinical characteristics between 25(OH)D concentration quartiles (Table 1). The quasi-linear trend between increase in the 25(OH)D concentration quartile and a decrease in all-cause in-hospital death and one-year readmission, as well as in mRS score on admission and at discharge was revealed. Moreover, with a switch to a higher quartile of serum 25(OH)D concentration, we also observed a quasi-linear increase in ejection fraction in echocardiography, as well as in the percentage of patients who fulfilled the criteria of efficient brain reperfusion after EMT (≥2b in modified Thrombolysis In Cerebral Infarction [mTICI] classification) and who at discharge achieved a score of no more than 2 on the mRS scale (i.e., no more than slight disability). Significant differences in the prevalence of cardiovascular and ischemic stroke risk factors, other than hypertension, were also found across the respective 25(OH)D quartiles. These relationships were U-shaped for diabetes mellitus, and L-shaped for chronic kidney disease.

Serum 25(OH)D concentration weakly correlates with mRS scores on admission (R = 0.17; *p* < 0.001), at discharge (R = 0.19; *p* < 0.001), and with achieving a score of no more than 2 on the mRS scale at discharge (R = 0.15; *p* < 0.001).

In Table 2, we present the relationships between the 25(OH)D quartiles and selected biochemical determinations, as well as single and composed indices of inflammatory response to brain injury determined on admission to the Neurology Department. With an increase in the 25(OH)D quartile, what we found was as follows: a decrease in neutrophil count and a decrease in blood glucose, HbA1c, CRP, and CRP-to-albumin, -lymphocyte, -neutrophil, -monocyte, and -platelet ratios; lower neutrophil-to-lymphocyte ratio (NLR); and a decrease in SII, SIRI, and NPS values (Table 2). Moreover, with an increase in the serum 25(OH)D concentration quartile, an increase in serum total and HDL cholesterol levels, as well as in albumin concentrations, was noted. Of the indices of the studied inflammatory response to brain injury, the strongest, although weakly statistically significant, rank Spearman’s correlations of the 25(OH)D quartiles were noted for the following: CRP-to-lymphocyte ratio (R = −0.15; *p* < 0.001), neutrophil-to-lymphocyte ratio (R = −0.13; *p* < 0.001), IBI (R = −0.17; *p* < 0.001), and SIRI (R = −0.14; *p* < 0.001).

In ROC analysis, we found only poor predictive power between serum 25(OH)D concentration and all-cause in-hospital mortality (cut-off: 20.48 ng/mL; area under the curve [AUC], 95% confidence interval [CI]: 0.587, 0.532–0.642; *p* = 0.002); readmission within 365 days after discharge (cut-off: 19.71 ng/mL; AUC, 95% CI: 0.545, 0.514–0.577; *p* = 0.005); and low disability score at discharge, of mRS ≤ 2 (cut-off: 46.86 ng/mL; AUC, 95% CI: 0.409, 0.357–0.467; *p* = 0.002). However, a serum 25(OH)D concentration equal to or greater than 46.86 ng/mL concerned only 97 (7%) of the patients studied.

In univariate logistic regression, we only obtained a statistically significant equation for serum 25(OH)D concentration with regard to one-year readmission occurrence (odds ratio [OR], 95% CI: 1.01, 1.00–1.002; *p* = 0.02). Patient functional status at discharge expressed by an mRS score ≤ 2 had no significant relation with continuous serum 25(OH)D concentration in univariate logistic regression; however, it reached a statistically significant association when serum 25(OH)D level was categorized as a quartile (OR, 95% CI: 1.41, 1.00–1.02; *p* = 0.02). The equation obtained suggests that switching by one quartile of 25(OH)D level increased the probability of a low disability score (mRS ≤ 2) at discharge by 41%. In multifactorial logistic regression (Chi^2^ = 43.64; *p* < 0.001), the quartiles of 25(OH)D level were the only independent variable with a positive influence on patient functional status (mRS ≤ 2) at discharge (OR, 95% CI: 1.52, 1.09–2.12; *p* = 0.012), along with a negative influence on mRS ≤ 2: neutrophil-to-lymphocyte ratio (OR, 95% CI: 0.89, 0.82–0.97; *p* < 0.01), age (OR, 95% CI: 0.96, 0.93–0.99; *p* = 0.003), comorbidity with diabetes mellitus (OR, 95% CI: 0.67, 0.26–1.74; *p* = 0.41), and treatment with EMT (OR, 95% CI: 0.27, 0.13–0.56; *p* < 0.01).

## 4. Discussion

In our retrospective analysis of the medical documentation of 1381 AIS patients, we found favorable associations between higher serum 25(OH)D concentration and AIS course, and prevalence of cardiovascular and AIS risk factors, such as diabetes mellitus, and chronic kidney disease (Table 1). Some relationships were quasi-linear (e.g., all-cause in-hospital mortality, readmission within one-year, mRS score at admission and discharge or percentage of patients with low disability score at discharge, expressed by mRS ≤ 2 score) and some quasi-L-, J- or U-shaped (e.g., age and diabetes mellitus) (Table 1). We also show that less than 25% of our AIS patients had an optimal 25(OH)D status, which, according to work by Fu et al. [1], amounts to >30 ng/mL (75 nmol/L). It has been accepted that 25(OH)D levels of 20–30 ng/mL (50–75 nmol/L) are considered “insufficient,” and levels of <20 ng/mL (<50 nmol/L) are considered “deficient” [1]. Interestingly, the ROC analysis indicated that the 25(OH)D level predicting all-cause in-hospital mortality in AIS patients was lower than that determined for a low disability score (mRS ≤ 2) at discharge (<20.48 vs. >46.86 ng/mL). On the one hand, this may be important for the dosing and monitoring of vitamin D supplementation according to its intended purpose, as well as for the design of future studies and the planning of outcome measures.

Our results corroborate the outcomes reported by other authors which show low serum 25(OH)D concentration as a biomarker of poor prognosis in AIS patients in regard to all-cause in-hospital mortality [15,25], readmission risk within one year after discharge (which can be regarded as equivalent to AIS recurrence risk) [18,20], and disability score at discharge [6,7,10,14,17,19,22,27], as well as poorer general health status (Table 1, multivariate logistic regression), and higher inflammation level (Table 2). Our study also corroborates a reported prevalence of 25(OH)D deficiency in AIS patients that was also raised by Kaul and Manikinda [5] and Ojaroodi et al. [36], who highlighted that as many as 86–89% of AIS patients were found to have deficient levels of 25(OH)D. Contrary to studies by Zhuo et al. [6] and Simon et al. [40], we did not observe a relationship between 25(OH)D concentration and NIHSS score. In our study we also did not confirm the relationships between serum 25(OH)D level and TOAST presumed etiological stroke categorization; however, we also found no publication which showed that low vitamin level is associated with single AIS subtype.

The clinical importance of the observation obtained seems only to lie in the confirmation of the use of 25(OH)D as a weak prognostic biomarker. According to Fu et al. [1], numerous studies on the effectiveness of 25(OH)D supplementation in order to prevent, ameliorate the course of, reduce disability score following, and discourage recurrence of AIS have failed to provide evidenced cardiovascular and functional benefits of 25(OH)D supplementation in AIS patients. This failure was probably due to enrolling participants with a baseline serum 25(OH)D concentration of >50 nmol/L [41], as well as the heterogeneity of participants’ baseline status with regard to cardiovascular risk, underlying comorbidities and general health status, type of treatment (e.g., medical, IVT, and EMT) [42], outcomes measured (e.g., primary and/or secondary AIS prevention, AIS treatment, and rehabilitation support), dosing regimens, intervention timing, genetic variability [35,43], and target serum concentrations of 25(OH)D [11,30]. However, some authors have argued that such supplementation might be effective if limited only to patients with low serum 25(OH)D concentration. Moreover, vitamin D supplementation ought to be administered as early as possible and in special dosing and timing, such as a sub-form of rapid treatment with 50,000 IU orally once a week for 6–8 weeks followed by 800 IU daily [5], or, as in the study by Hesami et al. [34], as the intramuscular administration of a single dose of 600,000 IU of vitamin D. In addition, the supplementation of vitamin D has to be associated with asking patients about the use of other dietary supplements (e.g., calcium) and comorbidities to prevent the overdosing of vitamin D observed in 10–33% of adult patients [5,44].

Moreover, to the best of our knowledge, our study is the first to identify associations between serum 25(OH)D concentration and values of known and validated indices of inflammatory response to brain injury [3], such as the CRP, neutrophil-to-lymphocyte ratio (NLR), CRP-to-platelet ratio, SII, SIRI, and NPS (Table 2). This observation might suggest the real-world importance of 25(OH)D activity as a modulator of systemic, and probably cerebral, inflammatory response to AIS [3,4,35]. However, the power of the associations observed was weak and with low determination coefficient (R^2^) values. The relationships showed that serum 25(OH)D concentration determines only 1.7–3.6% of variances in the indices mentioned. This observation only weakly corroborates the results of a study in rats showing vitamin D as a strong immunomodulator mitigating the influence of brain ischemia/reperfusion-induced systemic and local inflammation [35,45]. Nevertheless, we confirmed in multifactorial logistic regression, known from the literature [27], the importance of serum 25(OH)D concentration as an independent factor increasing the probability of a low disability score (mRS ≤ 2) at discharge on average by 52% for every quartile (Q1 → Q4), along with the negative effect of the neutrophil-to-lymphocyte ratio, a known and validated index of systemic inflammatory response to brain injury [3,4], older age, diabetes mellitus, and treatment with EMT as a source of ischemia/reperfusion-induced neuroinflammation.

As mentioned above, in our study we also found some statistically significant associations between serum 25(OH)D quartiles and cardiovascular and AIS risk factors [11,12,13,25,35,41], such as diabetes mellitus, blood glucose, and HbA1c (Table 1 and Table 2). The relationships between serum 25(OH)D quartiles and plasma lipid concentrations were statistically significant but appear to be of limited clinical importance (Table 2). Such unfavorable associations between low serum vitamin D concentration and the prevalence of diabetes mellitus [25,41], hypertension [35], and atrial fibrillation [35,36,37,38,39] have been reported previously. They can be explained by previously reported associations between low vitamin D levels and obesity and related metabolic disorders, as well as increased insulin resistance, endothelial dysfunction, oxidative stress, chronic inflammation (elevated hs-CRP, CRP, IL-6, and TNF-α), and dysregulation of the renin–angiotensin–aldosterone system [35]. On the one hand, these observations and pathomechanism showed that low vitamin D status may act through an increase in comorbidities, and, on the other hand, may reflect poor general health status being cause of higher mortality.

### Study Limitations and Strength

It is important to recognize that our study has a few limitations that need to be identified and addressed in future investigations. First, this study was conducted in a single center in Poland and the lack of an external validation study cohort may limit the generalizability of our findings to other ethnic and geographic populations, associated with potential confounders, including sun exposure, seasonality, socioeconomic status, general health and nutritional status, liver and kidney function, pre-stroke physical activity, vitamin D supplementation, comorbidities, changes in vitamin D level due to acute sickness, differences in time of blood sampling in regard to stroke onset due to delay in patient admission, transport, and endovascular treatment (EMT), etc. [35]. Moreover, this study was designed as a retrospective analysis of medical documentation, which should be recognized as an important study limitation even though it provides real-world data. One important potential limitation of such an analysis is, e.g., recall bias regarding vitamin D supplementation before admission, although we documented pre-stroke medical treatment and supplementation on the basis of both the medical interview (with patient or his or her family members) and the patient’s medication list. Therefore, future multi-center prospective studies with external validation are warranted to verify the broader clinical applicability of our results. Second, we only collected baseline levels of 25(OH)D and indices of inflammatory response to brain injury on admission, which were also not validated by determination of any cytokines, immune cell phenotyping, oxidative stress markers. The lack of serial (dynamic) measurements of 25(OH)D and inflammatory indices, as well as lack of neurological follow-up, made it impossible to monitor changes in inflammatory indices over time and identify which is the strongest in relation to admission serum vitamin D concentration, in order to determine the role of serum vitamin D concentration as a systemic modulator and, therefore, perhaps as a modulator of cerebral inflammation. Third, the outcome assessment in this study was limited to short-term all-cause mortality, and functional status at discharge only, without longer-term follow-up data (e.g., three months or one year). This relatively brief follow-up period restricts our ability to explore the associations of serum 25(OH)D concentration with long-term functional recovery, mortality, or stroke recurrence beyond the acute phase, which per se may reduce vitamin D level.

Despite these limitations, the present study provides valuable preliminary evidence. To the best of our knowledge, this is the first study to identify relationships between serum 25(OH)D concentration and baseline values of indices of inflammatory response to brain injury, known—as with 25(OH)D—as predictors of short-term prognosis in AIS patients. Our findings may thus provide a novel candidate indicator for prognostic stratification, especially in regard to disability level at discharge. This may help in planning post-stroke rehabilitation and social support after discharge. Our study also identified target serum 25(OH)D concentrations associated with patient survival (>20.5 ng/mL) and with low disability at discharge (>47 ng/mL); the concentration associated with low disability exceeds the target level reported for cardiovascular event prevention [41]. On the other hand, it should be underlined that the AUC obtained in ROC analysis was low (0.587 for all-cause mortality, and 0.509 for mRS ≤ 2), which may also suggest that the determination of serum 25(OH)D concentration may not be a good biomarker of poor and favorable AIS treatment outcomes. Such statement is supported by the fact that serum vitamin D concentrations above determined cut-offs were associated with younger age and lower comorbidity load among patients with a better prognosis (Table 1).

## 5. Conclusions

Higher serum vitamin D concentration in AIS patients was associated with lower all-cause mortality, a lower level of disability at discharge from hospital, a shorter length of in-hospital stay, and a lower risk of readmission within one year (equivalent to a lower risk of stroke recurrence). The association may be explained by vitamin D activity controlling the severity of cardiovascular risk factors and mitigating the inflammatory response to brain injury, but also by lower prevalence of underlying comorbidities.

## Figures and Tables

**Table 1:** Clinical characteristics of patients studied in relation to serum 25(OH)D concentration quartiles (<10.16; 10.16–17.77; 17.78–29.00; ≥29.01 ng/mL).

Parameter	Q1 (n = 344)	Q2 (n = 344)	Q3 (n = 347)	Q4 (n = 346)	Overall *p*-Value F or Chi^2^ Test	*p* Q1–Q2	*p* Q1–Q3	*p* Q1–Q4
Age (years)	70.66 ± 12.68	68.97 ± 13.37	67.09 ± 13.56	70.04 ± 12.89	<0.01 F(3,1377) = 4.92	0.089	<0.01	0.523
Male gender (n, %)	127 (38.9)	153 (44.5)	163 (47.0)	125 (36.1)	<0.01 Chi^2^(3) = 12.58	0.044	0.007	0.829
TOAST AIS subtype (n, %): Cardioembolic Large-artery atherosclerosis Small-vessel occlusion Stroke undetermined etiology	110 (32.0) 100 (29.0) 34 (10.0) 100 (29.0)	128 (37.2) 106 (30.8) 38 (11.0) 72 (21.0)	101 (29.1) 115 (33.1) 62 (17.9) 69 (19.9)	104 (30.1) 111 (32.1) 38 (11.0) 93 (26.8)	0.002 Chi^2^(9) = 25.85	0.168 0.567 0.669 0.016	0.392 0.256 0.088 0.744	0.570 0.392 0.668 0.519
Length of in-hospital stay (days)	12.66 ± 9.17	10.54 ± 7.23	12.88 ± 19.72	9.78 ± 8.06	<0.001 F(3) = 5.51	0.139	0.850	0.012
Diabetes mellitus (n, %)	50 (14.5)	28 (8.1)	27 (7.8)	30 (8.7)	<0.01 Chi^2^(3) = 11.92	0.008	0.005	0.016
Atrial fibrillation (n, %)	31 (9.0)	22 (6.4)	24 (6.9)	26 (7.5)	0.590 Chi^2^(3) = 1.91	0.199	0.310	0.476
Hypertension (n, %)	99 (28.8)	88 (25.6)	85 (24.5)	83 (24.0)	0.474 Chi^2^(3) = 2.50	0.347	0.203	0.154
Chronic coronary syndrome (n, %)	10 (2.9)	8 (2.3)	13 (3.8)	7 (2.0)	0.525 Chi^2^(3) = 2.23	0.633	0.539	0.455
Ejection fraction (%)	58.89 ± 10.68	59.77 ± 8.32	59.74 ± 9.97	60.79 ± 8.38	0.234 F(3,1377) = 1.42	0.354	0.401	0.044
Ejection fraction < 40% (n, %)	14 (4.1)	7 (2.0)	14 (4.0)	10 (2.8)	0.326 Chi^2^(3) = 6.95	0.036	0.700	0.124
Chronic kidney disease (n, %)	60 (17.4)	34 (9.9)	36 (10.4)	35 (10.1)	0.004 Chi^2^(3) = 13.18	0.004	0.007	0.005
All-cause in-hospital death (n, %)	35 (10.2)	28 (8.1)	24 (6.9)	16 (4.6)	0.044 Chi^2^(3) = 8.08	0.356	0.126	0.005
Readmission within 14 days (n, %)	19 (5.5)	17 (4.9)	13 (3.8)	22 (6.4)	0.463 Chi^2^(3) = 2.57	0.732	0.267	0.643
Readmission within 30 days (n, %)	42 (12.2)	40 (11.6)	28 (8.1)	37 (10.7)	0.301 Chi^2^(3) = 3.66	0.814	0.071	0.533
Readmission within one year (n, %)	152 (44.2)	121 (35.2)	124 (35.7)	118 (34.1)	0.023 Chi^2^(3) = 9.51	0.016	0.023	0.007
Hospital-acquired infection (n, %)	17 (4.9)	8 (2.3)	14 (4.0)	12 (3.5)	0.061 Chi^2^(3) = 7.38	0.008	0.138	0.094
Intravenous thrombolysis (n, %)	26 (7.6)	42 (12.2)	36 (10.4)	29 (8.4)	0.160 Chi^2^(3) = 5.17	0.038	0.210	0.713
Endovascular mechanical thrombectomy (n, %)	69 (20.1)	104 (30.2)	76 (21.9)	71 (20.5)	0.004 Chi^2^(3) = 13.19	0.002	0.552	0.880
mTICI ≥ 2b (n, %)	55 (79.7)	85 (81.7)	59 (77.6)	71 (100)	0.185 Chi^2^(3) = 4.81	0.153	0.297	0.073
Height (cm)	165.19 ± 9.04	166.35 ± 10.46	167.67 ± 8.89	165.87 ± 8.25	0.213 F(3,1377) = 1.50	0.965	0.237	0.965
Body weight (kg)	76.95 ± 19.33	77.21 ± 19.42	76.63 ± 16.07	75.74 ± 14.56	0.927 F(3,1377) = 0.15	0.919	0.891	0.965
% of ideal body weight	128.63 ± 28.99	127.52 ± 27.29	125.33 ± 22.58	126.41 ± 22.54	0.783 F(3,1377) = 0.783	0.765	0.891	0.965
NIHSS score on admission	14; 12–18	13.5; 9.5–18	14; 8–18	12; 8–18	0.633 F(3,1377) = 0.57	0.866	0.849	0.981
NIHSS score at discharge	12; 8–14.5	8.0; 4–13	9; 3–12	7; 3–14	0.282 F(3,1377) = 1.29	0.477	0.540	0.948
Delta NIHSS score	0; −5.5–0	−1; −10–0	−1; −10–0	0; −7–0	0.563 F(3,1377) = 0.683	0.983	0.915	0.901
mRS score on admission	4; 2–4	3; 1–4	3; 1–4	2; 1–4	<0.001 F(3,1377) = 8.21	0.821	0.013	<0.001
mRS score at discharge	3; 1–4	2; 1–3.5	1; 1–3	1; 1–3	<0.001 F(3,1377) = 7.09	0.038	<0.002	<0.001
Delta mRS	−1; −1–0	−1; −1–0	−1; −2–0	−1; −1–0	0.528 F(3,1377) = 0.740	0.999	0.908	0.999
mRS at discharge ≤ 2 (n, %)	133 (42.9)	106 (62.1)	206 (63.7)	238 (72.1)	<0.003 Chi^2^(3) = 14.40	0.017	0.010	<0.001

Abbreviations: mRS = modified Rankin Scale; mTICI = modified Thrombolysis In Cerebral Infarction scale; NIHSS = National Institute of Health Stroke Scale. TOAST = Trial of Org 10172 in Acute Stroke Treatment. Data presented as mean ± standard deviation; median; interquartile range; and number of patients (n) and percentage (%); statistical significance of difference was assessed with unifactorial ANOVA F(degrees of freedom, d.f.) test with overall *p*-value and Bonferroni post hoc test or with Ch^2^(d.f.) for categorical variables.

**Table 2:** Biochemical parameters and inflammatory response to brain injury indices in respective quartiles of serum 25(OH)D concentration (<10.16; 10.16–17.77; 17.78–29.00; ≥29.01 ng/mL).

Parameter	Q1 (n = 344)	Q2 (n = 344)	Q3 (n = 347)	Q4 (n = 346)	Overall *p*-Value F or Chi^2^ Test	*p* Q1–Q2	*p* Q1–Q3	*p* Q1–Q4
25(OH)D (ng/mL)	6.33 ± 2.28	13.71 ± 2.22	23.00 ± 3.15	42.63 ± 14.38	<0.001 F(3,1377) = 1497.9	<0.001	<0.001	<0.001
Hemoglobin (g/L)	13.33 ± 1.88	13.60 ± 1.65	13.62 ± 1.75	13.36 ± 1.71	0.044 F(3,1377) = 2.72	0.253	0.154	0.999
Hematocrit (%)	39.56 ± 5.07	40.28 ± 4.47	40.12 ± 4.69	39.54 ± 4.72	0.085 F(3,1377) = 2.21	0.049	0.136	0.952
Platelets (G/L)	246.58 ± 98.64	247.34 ± 78.49	231.36 ± 72.50	237.08 ± 81.14	0.031 F(3,1377) = 2.96	0.911	0.100	0.809
White blood cells (G/L)	9.74 ± 3.72	9.34 ± 3.32	9.18 ± 3.70	9.02 ± 4.12	0.071 F(3,1377) = 2.23	0.992	0.301	0.071
Neutrophils (G/L)	7.70 ± 4.50	6.65 ± 3.88	6.74 ± 4.40	6.27 ± 3.45	<0.001 F(3,1377) = 5.55	0.025	0.056	<0.001
Lymphocytes (G/L)	1.81 ± 1.38	1.74 ± 1.14	1.74 ± 0.71	2.22 ± 3.86	0.138 F(3,1377) = 1.84	0.999	0.999	0.305
Monocytes (G/L)	0.89 ± 0.78	0.73 ± 0.76	0.76 ± 0.38	0.86 ± 1.74	0.201 F(3,1377) = 1.55	0.386	0.702	0.998
Eosinophils (G/L)	0.13 ± 0.17	0.13 ± 0.19	0.13 ± 0.14	0.14 ± 0.14	0.995 F(3,1377) = 0.02	0.868	0.874	0.998
Total cholesterol (mg/dL)	141.10 ± 54.32	142.92 ± 52.25	170.76 ± 102.91	151.79 ± 52.50	0.002 F(3,1377) = 4.90	0.991	0.004	0.931
HDL cholesterol (mg/dL)	44.88 ± 14.22	45.08 ± 15.25	49.55 ± 19.22	51.10 ± 14.77	0.020 F(3,1377) = 3.31	0.929	0.287	0.006
Non-HDL cholesterol (mg/dL)	103.36 ± 50.44	96.97 ± 42.45	111.26 ± 47.27	106.46 ± 50.84	0.264 F(3,1377) = 1.33	0.406	0.302	0.716
LDL cholesterol (mg/dL)	102.33 ± 46.07	110.43 ± 47.92	108.05 ± 47.36	100.13 ± 40.62	0.013 F(3,1377) = 3.63	0.136	0.658	0.514
Triglycerides (mg/dL)	123.69 ± 67.25	126.60 ± 79.82	145.84 ± 155.60	120.58 ± 69.82	0.092 F(3,1377) = 2.23	0.723	0.283	0.684
Glucose (mg/dL)	158.30 ± 88.32	142.76 ± 62.66	132.51 ± 51.08	132.05 ± 44.53	<0.001 F(3,1377) = 10.77	0.023	<0.001	<0.001
HbA1c (%)	7.19 ± 2.89	6.75 ± 1.70	6.31 ± 1.15	6.03 ± 0.92	<0.001 F(3,1377) = 8.84	0.487	0.005	<0.001
Creatinine (mg/dL)	1.04 ± 0.64	0.95 ± 0.31	0.98 ± 0.39	1.01 ± 0.42	0.060 F(3,1377) = 2.48	0.069	0.444	0.917
Albumin (g/L)	3.32 ± 0.55	3.47 ± 0.61	3.53 ± 0.67	3.53 ± 0.64	0.003 F(3,1377) = 4.63	0.154	0.016	0.016
C-reactive protein (mg/dL)	5.15; 1.55–13.45	3.20; 3.04–3.87	3.00; 1.10–9.80	2.30; 1.0–6.70	<0.001 Chi^2^(3) = 28.48	0.610	0.033	0.002
CRP-to-albumin ratio	2.11; 0.52–5.12	0.98; 0.38–3.55	1.37; 0.28–4.04	0.83; 0.37–2.80	0.006 Chi^2^(3) = 12.38	0.951	0.923	0.670
CRP-to-lymphocyte ratio	3.55; 1.14–9.48	2.08; 0.71–8.84	1.48; 0.71–6.18	1.48; 0.61–5.40	<0.001 Chi^2^(3) = 33.71	0.258	0.643	0.022
CRP-to-neutrophil ratio	0.90; 0.25–2.20	0.55; 0.20–1.86	0.54; 0.21–1.47	0.48; 0.21–1.22	0.002 Chi^2^(3) = 19.81	0.296	0.070	0.025
CRP-to-monocyte ratio	8.07; 2.43–19.84	4.84; 1.64–17.28	4.38; 1.67–13.94	3.82; 1.77–10.85	0.002 Chi^2^(3) = 14.61	0.252	0.186	0.021
CRP-to-platelet ratio	0.02; 0.01–0.06	0.01; 0.1–004	0.01; 0.01–0.05	0.01; 0.0–0.03	<0.001 Chi^2^(3) = 23.91	0.178	0.136	0.039
Neutrophil-to-lymphocyte ratio	4.00; 2.48–7.77	3.54; 2.08–6.44	3.15; 2.02–6.04	2.80; 1.95–5.95	0.002 Chi^2^(3) = 14.66	0.205	0.909	0.020
Neutrophil-to-platelet ratio	42.88; 28.00–75.57	45.65; 27.99–74.87	40.73; 27.82–57.08	35.64; 25.51–63.20	0.117 Chi^2^(3) = 5.89	0.878	0.278	0.070
Platelet-to-lymphocyte ratio	153.59; 98.53–230.28	159.32; 104.0–227.40	134.94; 101.62–197.13	131.89; 91.30–201.35	0.002 Chi^2^(3) = 14.76	0.820	0.129	0.053
Platelet-to-albumin ratio	69.61; 53.18–89.00	68.44; 53.26–84.91	62.08; 51.51–81.47	63.40; 48.70–83.43	0.185 Chi^2^(3) = 4.83	0.700	0.033	0.285
Lymphocyte-to-albumin ratio	0.48; 0.32–0.65	0.42; 0.30–0.57	0.45; 0.37–0.57	0.45; 0.29–0.71	0.324 Chi^2^(3) = 3.47	0.042	0.120	0.174
Neutrophil-to-albumin ratio	2.10; 1.41–3.28	1.71; 1.12–2.73	1.91; 1.11–3.05	1.68; 1.28–2.96	0.030 Chi^2^(3) = 8.93	0.030	0.439	0.033
Lymphocyte-to-monocyte ratio	2.17; 1.41–3.04	2.44; 1.57–3.35	2.59; 1.55–3.72	2.68; 1.63–3.75	0.002 Chi^2^(3) = 15.30	0.168	0.158	0.233
HLAN	10.33; 5.04–16.24	11.56; 5.91–20.08	9.53; 4.98–21.40	11.77; 17.19–50.60	0.621 Chi^2^(3) = 1.77	0.338	0.308	0.116
HALP	27.65; 17.45–44.49	25.99; 16.04–41.40	31.94; 19.91–50.52	30.23; 17.19–50.60	0.030 Chi^2^(3) = 8.96	0.542	0.057	0.061
SII	917.64; 559.74–1838.67	868.34; 467.25–1685.13	688.26; 410.58–1511.61	694.59; 384.52–1367.12	0.005 Chi^2^(3) = 13.02	0.134	0.640	0.005
SIRI	3.09; 1.60–6.84	2.23; 1.35–4.66	2.06; 1.10–4.91	1.85; 1.02–4.07	<0.001 Chi^2^(3) = 21.14	0.072	0.399	0.006
NPS	4.0; 3.0–4.0	3.50; 3.0–4.0	3.0; 3.0–4.0	3.0; 2.0–4.0	0.028 Chi^2^(3) = 9.65	0.332	0.195	0.009
IBI	21.85; 6.53–72.68	11.32; 3.52–56.86	8.16; 3.55–40.96	8.76; 2.85–31.99	<0.001 Chi^2^(3) = 38.22	0.140	0.860	0.034
CALLY	7.90; 2.82–31.96	13.20; 3.78–40.08	11.07; 3.46–50.84	18.20; 5.03–41.84	0.012 Chi^2^(3) = 10.93	0.220	0.215	0.017
CRP-to-HDL cholesterol ratio	0.06; 0.02–0.24	0.06; 0.03–0.15	0.04; 0.02–0.13	0.04; 0.02–0.12	0.013 Chi^2^(3) = 10.84	0.271	0.026	0.721
Monocyte-to-HDL cholesterol ratio	0.01; 0.01–0.02	0.01; 0.01–0.02	0.01; 0.01–0.02	0.01; 0.01–0.02	0.314 Chi^2^(3) = 3.55	0.981	0.770	0.081
Platelet-to-hemoglobin ratio	17.01; 13.50–21.64	17.61; 14.02–21.54	16.42; 13.40–20.24	16.38; 13.36–21.14	0.038 Chi^2^(3) = 8.42	0.439	0.005	0.142
NRI	91.14; 86.74–98.05	94.26; 87.88–100.49	93.65; 87.42–102.0	95.47; 88.94–103.22	0.005 Chi^2^(3) = 13.09	0.018	0.002	0.002
NPAR	21.96; 18.45–26.48	20.94; 16.89–25.12	21.42; 16.42–25.94	20.05; 16.82–23.79	0.114 Chi^2^(3) = 5.95	0.064	0.073	0.007

Abbreviations: CALLY = CRP–albumin–lymphocyte index; CRP = C-reactive protein; HALP = hemoglobin–albumin–lymphocyte–platelet index; HDL = high-density lipoprotein; HLAN = hemoglobin–lymphocyte–albumin–neutrophil index; IBI = Inflammatory Burden Index; NPAR = neutrophil percentage-to-albumin ratio; NPS = Naples Prognostic Score; NRI = Nutritional Risk Index; SII = Systemic Immune-Inflammation Index; SIRI = Systemic Inflammation Response Index. Notes: Data presented as median; interquartile range; statistical significance of difference was assessed with unifactorial ANOVA F (degrees of freedom, d.f.) test with overall *p*-value and Bonferroni post hoc test (for parametric variables), and Kruskal–Wallis ANOVA with Chi^2^ (d.f.) for non-parametric variables.

## Data Availability

The original contributions presented in this study are included in the article. The anonymized data supporting the findings of this study are available from the corresponding authors upon reasonable request, subject to institutional data-sharing policies. The data are not publicly available due to privacy and ethical restrictions.

## Abbreviation

25(OH)D	vitamin D
CALLY	CRP-albumin-lymphocyte
CRP	C- reactive protein
EMT	endovascular mechanical thrombectomy
HALP	hemoglobin-albumin-lymphocyte-platelet
HbA1c	hemoglobin A1c
HDL	high- density lipoprotein
HLAN	lymphocyte-albumin-neutrophil
IBI	Inflammatory Burden Index (CRP, NLR)
IVT	intravenous thrombolysis
LDL	low-density lipoprotein
LMR	lymphocyte-to-monocyte ratio
LOS	length of in-hospital stay
mRS	modified Rankin Scale;
mTICI	modified Thrombolysis In Cerebral Infarction scale;
NIHSS	National Institute of Health Stroke Scale.
NLR	neutrophil-to-lymphocyte ratio
NPAR	neutrophil percentage-to-albumin ratio
NRI	The Nutritional Risk Index
PHR	platelet-to-hemoglobin ratio
rt-PA	recombinant tissue plasminogen activator
SII	Systemic Immune-Inflammation Index
SIRI	Systemic Inflammation Response Index
TOAST	Trial of Org 10172 in Acute Stroke Treatment
CE	cardioembolic stroke
LAA	large artery atherosclerosis
SVO	small vessel occlusion
SUE	stroke of undetermined etiology
